# Fracture resistance of fiber post in endodontically treated teeth

**DOI:** 10.6026/973206300213847

**Published:** 2025-10-31

**Authors:** Gahana G.C, Chaithanya Ramisetty Nagabhushan, Srinidhi Bhat, Jaya Krishna T, Harsha Suresh Hegde, Vishnukumara M

**Affiliations:** 1 Department of Prosthodontics, Sharavathi Dental College & Hospital, Shimoga, Karnataka, India; 2 Department of Prosthodontics, KVG Dental College & Hospital, Sullia, Karnataka, India

**Keywords:** Everstick post, tenaxtrans fiber post, angelus reforpost, maxcem elite, GC fujicem, fracture resistance

## Abstract

Management of an endodontically treated tooth is a challenging task. Post and core procedure helps to enhance the fracture resistance of endodontically treated teeth. Hence the present study was done to evaluate the fracture resistance of custom made GC Everstick post to prefabricated Tenax fiber post and Angelus Reforpostluted with two different resin cements on endodontically treated teeth. Sixty extracted human mono-radicular teeth were included. They were endodontically treated and standardized to 15mm from the root apex and then decoronated. The specimens were randomly divided into 3 groups and each group was further alienated into 2 subgroups. The teeth were randomly selected and treated with an Everstick post, Angelus reforpost, Tenax trans fiber post and were cemented using Maxcem elite and GC Fujicem cement. The core was standardized to 4mm. All the Samples were thermocycled and embedded in the acrylic resin blocks then subjected to compressive load of 1mm/min at 1350 angulation to evaluate the fracture resistance. Thus, we show that the GC Everstick Post showed better fracture resistance when compared to other post irrespective of the resin cement used.Maxcem elite resin cement provided better fracture resistance.

## Background:

Management of an endodontically treated tooth is a challenging task for a prosthodontist, as the stability and retention of the restoration are compromised which increases the probability of fracture of the tooth during function [1]. Hence to provide adequate stability and retention to the restoration inter radicular posts were introduced. There are different types of posts, custom cast posts and prefabricated posts, based on the designs; parallel/tapered, smooth/serrated/threaded [2]. Prefabricated posts are further classified depending on the type of material used zirconia, ceramic, fiber reinforced resin, metal posts [2]. In order to lower the danger of coronal fracture, the post's function is to shift stresses downward into the root while maintaining a core repair and crown [3, 4]. The chances of survival of an endodontically treated tooth depend on the quality and quantity of remaining dental tissue. However, dentin must be removed during canal preparation and the subsequent reduction in fracture resistance may compensate any likely gains. The post does not strengthen the root, but rather serves to improve retention of the core [5]. According to the theory of mechanics, a post and core system with a high modulus of elasticity-such as a metallic and stainless steel post-transfers stress from the rigid post to the less rigid dentin, causing the remaining teeth to fracture as a result of the excessive stress transfer from the post. In order to address this issue, posts with dentin-like elastic moduli and the growing desire for aesthetics have led to the creation of metal-free post-and-core systems, or fibre post systems, which are tooth-colored and reflect and transmit light in a manner similar to that of natural teeth [6, 7]. Better aesthetic qualities are seen because of their translucency, and just a small quantity of dentin is removed during canal preparation, maintaining root structure [8, 9]. Because the dentist completes every stage in a single visit, prefabricated fibre posts streamline restorative operations [8]. Prefabricated Angelus Reforpost and Tenax trans fiber post are such popular tooth-colored glass fiber posts used in the present study. Following the development of the fibre, a new soft, flexible, individually formable resin-impregnated E-glass fibre post (Everstick Post, GC) with an inter-penetrating polymer network was recently introduced to accommodate any root canal morphology. It has been determined that sticky resin cements are a superior choice for the restoration of teeth that have undergone endodontic treatment [9]. In addition to dramatically reducing the number of application steps, recently developed self-adhesive resin cements eliminate the need for preparation of the dentin, which shortens clinical treatment times and reduces method sensitivity. Evaluation of fracture resistance is important since it is the most commonly seen in endodontically treated teeth. Hence, the current *in vitro* study was designed to compare and assess the fracture resistance of custom made GC Everstick post to prefabricated Tenax fiber post and Angelus Reforpostluted with two different resin cements in an endodontically treated tooth and also to give the clinician an idea regarding their mechanical properties.

## Materials and Methodology:

Overviews of the materials used in this research are illustrated in Table 1 (see PDF). Everstick post (GC-Europe), Tenax Trans fiber post (Coltene, Whaledent, USA), Angelus Reforpost (Angelus, Delhi, India), Maxcem elite (KERR-Sybro USA), GC Fujicem (Gc-Europe), Paracore (Coltene, Whaledent, USA) and a metal mold to fabricate resin blocks were utilized (Table 2 - see PDF). The study consisted of 60 extracted single rooted teeth. The selection of the specimens was based on the following inclusion criteria: (a) Intact straight single rooted teeth; (b) Single root canal; (c) Teeth with complete root formation / closed apex; (d) Teeth free of caries; and (e) Teeth with no anatomic variations. The presence of teeth with extremely curved roots, fractured roots, calcified roots and root caries were excluded from the study. 60 extracted human single rooted teeth were inlcuded as per inclusion and exclusion criteria and were Root canal treated. The Root lengths was standardized for all the specimens at 15mm from the root apex and with the help of a slow speed diamond disc, decoronation of all the teeth was performed. The post space was standardized to the depth of 10mm, leaving 5mm of intact Gutta-Percha to preserve the apical seal. The post space preparation was done using the tissue saving principle with special preparation drills according to the manufacturer's instruction for all the teeth. The Everstick post with a diameter of 1.5mm was selected. The post was shaped according to the root canal space, light cured. Later, the surface of the post was activated using a resin adhesive, post was under a light shield for 3 to 5 minutes and then light cured thoroughly for 10 sec. The TenaxTrans fiber post and Angelus Reforpost of 1.5mm diameter was selected and the drills were used according to the manufacturer's instruction. 3mm of the post was left outside to reinforce the core. The groups 1A, 2A and 3A, Maxcem elite resin cement which is a "Self-adhesive" luting agent was used to lute the post into the root canals whereas the groups 1B, 2B and 3B, GC Fujicem resin cement was used to lute the post into the root canals according to the manufacturer's instruction. After post cementation core build-up done using composite resin (ParacoreColtene). The core height was standardized to 4mm for all the specimens. The specimens were mounted on the Universal testing machine and compressive load was applied to the tooth at an angle of 135 degrees as presented in Figure 1A (see PDF), at a cross-head speed of 1mm/min until fracture occurs. The load at which fracture occurred was recorded and expressed in Newton. The location of fracture was classified under Stereomicroscope (40x) after mechanical testing. The location of the fracture was noted as favourable and catastrophic. Core fracture and root fractures at the cervical third was classified as favourable (repairable). Root fractures at the middle and apical thirds were classified as catastrophic (not repairable). All the specimens showed favourable fracture. A statistical software G*power, version 3.0.1 (Franz Faul, universitat Kiel, Germany) was used to statistically assess the fracture resistance of three different fiber post system cemented with two different resin cements in endodontically treated teeth. The outcomes of the Independent sample t test indicated that the p- values were of statistical significance.

## Results:

The mean and standard deviations of the fracture resistance are outlined in Table 3 (see PDF) and Figure 1B (see PDF), 1C (see PDF) & 1D (see PDF). The results of the Independent sample t test showed that in group 1 and group 3 there is a significant difference (p< 0.001) in the fracture resistance between the subgroups 1A, 3A that were cemented using Maxcem Elite resin cement and 1B, 3B that were cemented using GC Fujicem resin cement. When Group 1A was compared with Group 1B, the mean difference was found to be 146.51 (p<0.001), when 3A is compared with Group 3B, the mean difference was found to be 135.58 (p<0.001), which is statistically significant. When Group 2A is compared with Group 2B, the mean difference was found to be 16.23 and the p value is 0.34 which is statistically not significant.

## Discussion:

The present *in vitro* study was conducted to assess the fracture resistance of three different fiber post system cemented with two different resin cements in endodontically treated tooth. The restoration of endodontically treated teeth is a major concern in dentistry although it's been practiced for many years [1]. Decreased moisture content, loss of tooth structure from dental caries, fracture of previous restoration being the problem, hence post and core was introduced. In order to lower the likelihood of coronal fracture, the post's function is to transmit stresses downward onto the root while maintaining a core and crown [3, 4]. Post and core systems have their own advantages and disadvantages, varying from custom cast post to prefabricated posts. For many years, the cast post and core procedure has been regarded as the gold standard for restoration, [10] because of the successful clinical service. Fibre posts can be suggested as a superior substitute for cast posts as Agarwal *et al.* [10] and Rosentritt *et al.* [11] found that the fracture resistance of teeth restored with fibre posts will be on par with or higher than that of teeth restored with metal posts. Since their inception, some researchers have examined the fracture resistance of fibre posts and contrasted them with metal posts and other prefabricated fibre posts; sadly, the findings have not always been consistent. For many years, the cast post and core procedure has been regarded as the gold standard for restoration [12]. Some investigators claim that parallel and serrated posts are more retentive than the conical ones and achieve a more uniform and minimal distribution of stress in function, [13] shows a lower frequency of fracture upon failurehence Tenax Trans fiber post and Angelus Reforpost are the prefabricated posts selected for the present study [1, 3]. Ever Stick™ Post claims to be a novel tooth rehabilitation material which is an adjust table, soft, flexible and unpolymerized glass fiber post that has the potential to adapt to the morphology of the root canal [14]. The etch-and-rinse method calls for removing the smear layer entirely with a separate etching phase, then washing the surfaces with water and letting them air dry before applying the adhesive system [15, 16]. By using resin cements, glued constructions can better withstand functional stresses, improve marginal adaptation with a better apical seal, increase the retention of short-length posts, and optimise fracture patterns when they do occur [17]. In the present study Maxcem elite self- adhesive resin cement showed higher fracture resistance compared to dual cure resin cement, this is in congruent with the study results of Gopal *et al.* [18] which might be due to limited ability to diffuse light across the entire length of resin cement, thereby compromising the degree of polymerization and conversion [9]. Hayashi *et al.* [19] (2006) studied the mode of fractures in teeth restored with fiber post and cast metal post-core, which were subjected to oblique and vertical load. The vertical load caused cracks propagating in the middle and apical portion of the roots while in the oblique load, fracture occurred in the cervical part of the root when fiber posts were used. Hence oblique load i.e., 1350 angulation was selected for the present study. In the present study to simulate the clinical situation, cyclic loading has been used, during loading compressive head was maintained at constant speed of 1mm/min at 1350 angulation. The maximum physiologic forces operating on the teeth in the oral cavity are less than the *in-vitro* pressures that cause failure, according to clinical observations [3]. However, given the current situation, fibre post can be suggested since it has excellent physical characteristics, such as a modulus of elasticity that is near to dentin, good fracture resistance, and the patient-desired aesthetics. However, before this is considered for clinical application, more extensive research on the subject is needed. For the long-term outcomes, more *in vitro* and *in vivo* research is needed.

## Conclusion:

GC Everstick showed better fracture resistance when compared to other post irrespective of the resin cement used. Maxcem elite resin (self-adhesive) cement provided better fracture resistance than GC Fujicem resin cement (dual cure) irrespective of the post used. Stereomicroscopic analysis of all the fractured specimens showed predominately favorable type of fracture with all the samples fracturing at core level. The results of this *in vitro* study need to be further validated in the clinical scenario. Furthermore, a clinical trial involving the following post systems and luting agents would be an apt extension to this study.

## References

[1] Fernandes AS (2001). Int J Prosthodont..

[2] Cheung W (2005). J Am Dent Assoc..

[3] Assif D (1993). J Prosthet Dent..

[4] Assif D (1994). J Prosthet Dent..

[5] Martinez AI (1998). J Prosthet Dent..

[6] Hayashi M (2008). Jpn Dent Sci Rev..

[7] deMoraes AP (2013). Appl Adhe Sci..

[8] Pereira JR (2014). J Appl Oral Sci..

[9] Silva RA (2011). J Appl Oral Sci..

[10] Aggarwal R (2013). J Oral BiolCraniofac Res..

[11] Rosentritt M (2000). J Oral Rehabil..

[12] Maroulakos G (2015). J Prosthet Dent..

[13] Chirila M (2021). J Med Life..

[14] Sinha S (2017). Int J Oral Health Dent..

[15] Akgungor G (2006). J Prosthet Dent..

[16] Burrer P (2020). Polymers..

[17] Schmitter M (2006). J Endod..

[18] Gopal S (2017). J Clin Diagn Res..

[19] Hayashi M (2006). Dent Mater..

